# Pulmonary miliary tuberculosis complicated with tuberculous spondylitis; an extraordinary rare association: a case report

**DOI:** 10.4076/1757-1626-2-7983

**Published:** 2009-09-08

**Authors:** Eleni Palama, Christos Golias, Iosif Illiadis, Konstantinos Charalabopoulos

**Affiliations:** 1 Department of Medicine and Pulmonary Diseases, Grevena State Hospital51100 GrevenaGreece; 2 Department of Physiology, Clinical Unit, University of Ioannina Medical Faculty13 Solomou Street 452 21 IoanninaGreece

## Abstract

In the last decade a significant rise in the prevalence of tuberculosis as well as in its extrapulmonary manifestations is detected worldwide. The central nervous system, the genitourinary tract, the organs of the abdomen and the skeletal system, are common sites of infection. Misdiagnosis and delay in treatment are common events. Herein, we present a case of a 78-year-old man non-smoker, with miliary tuberculosis complicated with tuberculous spondylitis. The patient presented with anemia and a left shoulder pain, accompanied by rigor and fever 37.5°C-38°C of one month duration. This entity is extremely rare, since only two similar cases have been reported in the English literature according to PubMed search.

## Introduction

During the last years it is more than evident that the prevalence of pulmonary and extrapulmonary tuberculosis (TB), mainly in western European countries, has risen significantly. The aetiology of this phenomenon is multifactorial. The main parameters are the development of drug-resistant strains of *Mycobacterium tuberculosis*, aging population demographics, massive refugee waves from high prevalence for tuberculosis disease territories, increase in the number of health care workers exposed to the disease and the rising number of people with immunosuppression [1,2]. Diagnosis of extrapulmonary tuberculosis is often difficult. The genitourinary system is a usual extrapulmonary location. The most common manifestation of abdominal tuberculosis is lymphadenopathy and ileocecal involvement is present in 80%-90% of patients. Central nervous system tuberculosis involvement has various manifestations, such as meningitis, tuberculoma, abscess, cerebritis, and miliary tuberculosis. Spinal tuberculosis accounts for approximately 50% of cases of skeletal tuberculosis [3]. The most common location is L1 (lumbar) vertebra although more than one vertebral bodies are typically affected. The disease process most often begins in the anterior part of the vertebral body adjacent to the end plate. Later on the disk space is involved via a number of routes. Pott’s disease, is a rare presentation of extrapulmonary tuberculosis that affects spine, vertebrae and intevertebral joints. The disease is named after Percivall Pott (1714-1788) description, a surgeon in London, U.K. The disease is also called tuberculous spondylitis and is mostly localized in the thoracic portion of the spine. Herein, we present a case of miliary tuberculosis complicated by tuberculous spondylitis, which was confronted by administration of regressive treatment before the establishment of the definite diagnosis.

## Case presentation

**Figure 1.**
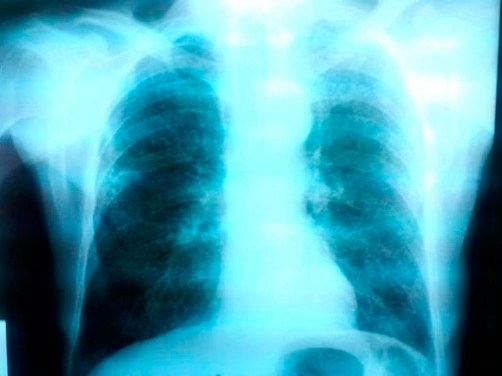
Postero-anterior chest radiograph revealing multiple diffuse small opacities at both lungs.

**Figure 2.**
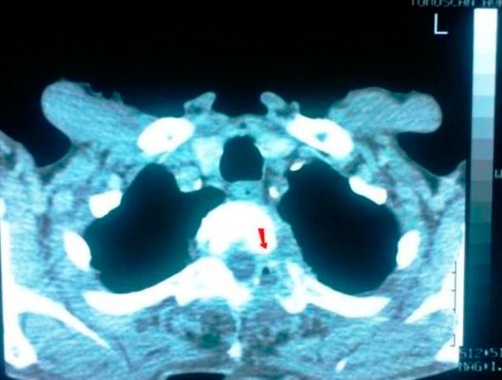
CT scan of the thoracic cage-vertebrae showing (arrow) mass of smooth consistency laterally to the left at the level of T_2_ (thoracic) vertebra accompanied by osteolytic damage (erosions) of the vertebral bodies.

**Figure 3.**
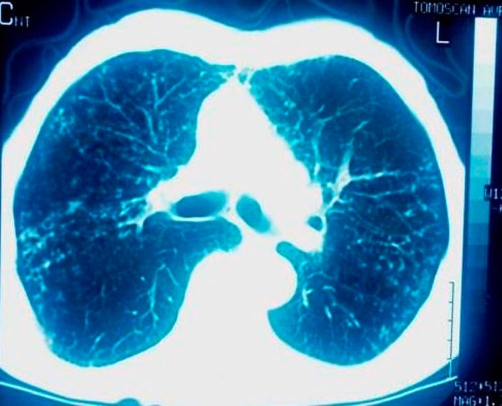
CT scan image of the pulmonary parenchyma showing multiple bilaterally diffused opacities giving the picture of ‘bud in tree’.

**Figure 4 and 5.**
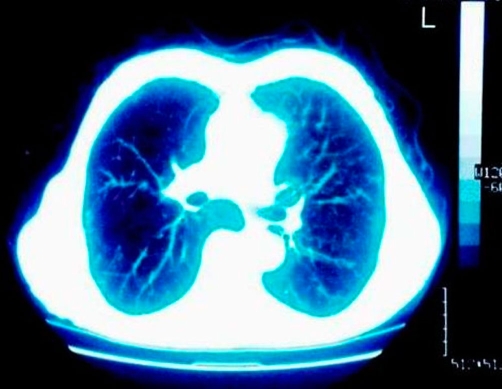

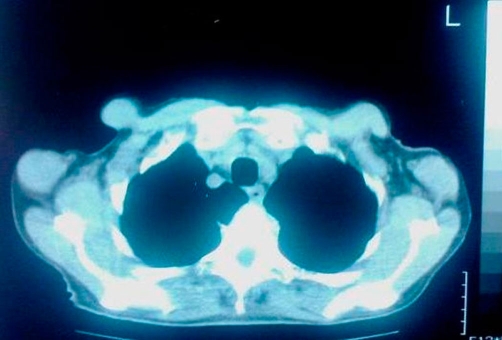
CT of the thorax after one year treatment revealing a healthy patient.

A 78-year-old Caucasian man of Greek origin, 80 kg in weight, 174 cm in height, non-smoker, was referred to Grevena State Hospital, Greece, complaining for a left shoulder pain, which was aggravated by movement. He also reported rigor and fever between 37°5 and 38°C, persisting for one month. Ten days before his first admission, he was examined by an orthopaedic surgeon and had received analgesic-anti-inflammatory drugs without any clinical improvement. From his personal medical history, the patient reported multiple medical visits complaining of persistent cough and abundant purulent sputum without fever for years. Due to these symptoms he was subjected to two CT scans of the thorax and one fiberoptic bronchoscopy without any conclusive results. For the last two years, he was thought to suffer from episodes of chronic bronchitis. On admission, a postero-anterior chest radiograph was performed which revealed multiple diffuse small opacities at both lungs (Figure 1), which prompted further investigation. Physical examination revealed temperature 38.3°C, heart rate 100 beats/min, 18 inspirations/min, and blood pressure 110/60 mmHg. Auscultation of the lungs was not conclusive; some fine crackles were audible. White blood cell (WBC) count was 16.2 × 10^9^/L, with 86.3% neutrophils, 4.3% lymphocytes, 7.7% monocytes, 1.6% eosinophils). Platelets (PLT):512000/mm^3^, haematocrit (Ht):29.1%, erythrocyte sedimentation rate (ESR) 102 mm/h and C-reactive protein 20.97 mg/dl (normal value < 0.8 mg/dl). Biochemical tests revealed increased transaminases with serum glutamic oxaloacetic transaminase (SGOT): 132 IU, serum glutamic pyruvic transaminase (SGPT): 90 IU and hypoalbuminaemia with serum albumins 2.30 g/dL, total protein 5.90 g/dl and lactate dehydrogenase (LDH): 149 IU/L. Urine test was normal. Tuberculin skin reaction was negative. Consecutive blood cultures were negative. Chest computer tomography (CT) showed lymphadenopathy of the mediastinum, a mass of smooth consistency laterally to the left at the level of T_2_ (thoracic) vertebra, accompanied by osteolytic erosions of the vertebral bodies of the second to the fifth thoracic vertebrae (Figure 2). Additionally, multiple bilaterally diffused opacities giving the picture of ‘bud in tree’ (Figure 3) and a very small bubble filled with air, were revealed. The radiologists’ diagnosis was miliary tuberculosis complicated with tuberculous spondylitis accompanied by a small lung abscess. The abdomen and cerebral CT were normal. Gram stain and cultures for common bacteria were taken from sputum secretions. They were negative for *Mycobacterium tuberculosis*. Bronchoscopy one month later was negative for endobronchial mass and non revelatory for tuberculosis; however, an atrophy of the mucosa was noted. On cytology, bronchial aspirate was negative at cultivation for Koch’s bacillus; a polymerase chain reaction (PCR) was also negative. Antituberculous chemotherapy, with isoniazid, rifampicin, and ethambutol, was initiated from the first day of admission. After 3 days of hospitalization, a repeated chest CT revealed improvement of findings. Clinical improvement was noted after the first week of therapy. Ht increased towards normal limits and ESR was significantly decreased. Transaminasemia was diminished. The patient after his discharged followed the anti-TB therapy for one year. During this period, he was followed up periodically by a pneumonologist. Four months later, chest radiograph and thoracic CT showed a significant improvement. On the 12^th^ month of therapy, thoracic CT revealed a complete remission (Figures 4 and 5).

## Discussion

Miliary tuberculosis (or disseminated TB) is a form of tuberculosis which is characterized by wide dissemination into the human body and by the tiny size of the lesions (1-5 mm). It is caused by the widespread hematogenous dissemination of *Mycobacterium tuberculosis*. Classic miliary TB is defined as millet-like (mean 2 mm, range 1-5 mm) seeding of TB bacilli in the lung, as evidenced on chest radiography. Miliary TB may occur in an individual organ (very rare, <5%) or may affect any number of organs throughout the whole body (>90%), including brain, lungs, liver, and spleen. It is a complication of 1-3% of all TB cases [4].

Miliary TB may mimic many diseases. Symptoms including fever, night sweats, weight loss, hematologic abnormalities, cough, pleurisy, dyspnea, and hemoptysis are not necessarily specific. In some case series, up to 50% of cases are not diagnosed antemortem. If untreated, the mortality rate is assumed to be close to 100%, but with early and appropriate treatment, the mortality rate is reduced to less than 10%. Therefore, a high index of clinical suspicion is important to obtain an early diagnosis and to ensure improved clinical outcomes [4]. Early empirical treatment for possible but not yet definitive miliary TB increases the likelihood of survival and should never be withheld while test results are pending.

Skeletal tuberculosis comprises 35% of cases presenting extrapulmonary tuberculous manifestations. Skeletal tuberculosis includes entities such as spinal tuberculosis (Pott’s disease), articular tuberculosis, Poncet’s disease (rare, acute sterile polyarthritis associated with visceral involvement) and tuberculous osteomyelitis.

Tuberculosis of the spine is an uncommon form of tuberculosis occurring in less than 1% of patients with tuberculosis [5,6]. Typical radiographic changes indicative of Pott’s disease include vertebral destruction and narrowing of the intervertebral space seen on plain radiographs. Correlative CT and magnetic resonance imaging (MRI) could aid substantially in the diagnosis of spinal TB due to their ability to show lytic lesions and adjacent abscess formation. CT scan could be considered the most useful radiologic tool both for the diagnosis and determining the extent of the disease. In our patient CT scan revealed positive findings such as anterior vertebral body destruction and paraspinal abscess. In addition, the diagnosis may be confirmed by CT guided needle aspiration biopsy. The samples obtained are assessed with stains for acid fast bacilli, culture and histological examination. It is essential prompt diagnosis to be established and early treatment to be administered in order to prevent neurological deficit and irreversible damage [7,8].

Spinal tuberculosis is a chronic and slowly progresive disease with prolonged symptomatology. The clinical history, as well as the presenting condition of the patient, are important but not always reliable for an immediate diagnosis. Pain is the most common presenting symptom. It usually has an insidious onset and may be mechanical in nature during the initial stages of the disease. Systemic symptoms arise with progression of disease. Persistent spinal pain and local tenderness, limitation of spinal mobility, febrile state, and neurologic complications including paralytic phenomena, present as the destruction continues. Other symptoms are reflective of chronic illness including malaise, weight loss, and fatigue [5]. Diagnosis is frequently not suspected in patients with no evidence of extraspinal tuberculosis. The clinical presentation together with the radiologic appearance of the spine and a positive tuberculin test may suggest spinal tuberculosis. The diagnosis must be confirmed by evidence of acid-fast bacilli from the bone or body fluids. To our best knowledge, association of miliary tuberculosis and Pott’s disease is rather rare with only two cases being reported in the English literature [8,9]. The physician could easily miss the diagnosis since this association presents a rare clinical entity.

Our patient had negative tuberculin skin test as well as negative sputum cytology and cultivation for Koch’s bacillus after bronchoscopy although he presented pertinacious clinical signs and had rather suspicious radiological findings. The clinical and radiologic features of extrapulmonary tuberculosis may mimic those of many diseases. A high index of suspicion is required, especially in high-risk populations. Although a positive chest radiograph or positive tuberculin skin test supports the diagnosis, negative results do not exclude the possibility of extrapulmonary tuberculosis [10].

Anti-TB therapy can minimize morbidity and mortality but it may be initiated empirically. A negative smear for acid-fast bacillus, the lack of granulomas on histopathology, and failure to culture *Mycobacterium tuberculosis* do not exclude diagnosis. Novel diagnostic modalities such as adenosine deaminase levels and polymerase chain reaction method can be useful in certain forms of extrapulmonary tuberculosis.

In general, the same regimens are used to treat pulmonary and extrapulmonary tuberculosis, and responses to antituberculous therapy are similar in patients with immunosuppressive disease as well as in those without. Our patient proved to be a potentially easily misdiagnosed case since most of the basic laboratory findings regarding tuberculosis were negative. Nevertheless, an empirical initiation of anti-TB treatment based on his previous and present clinical manifestations was proved quite beneficial while more extensive laboratory tests such as CT imaging and CT guided needle aspiration biopsy were pending.

At present, the treatment of Pott’s disease remains controversial. Some advocate conservative treatment with late spinal fusion and others early spinal fusion followed by conservative treatment. Surgical treatment should include anti-TB medication and abscess decompression [11-13]. Chemotherapy remained the cornerstone in our patient. During his follow up there was strong evidence of significant improvement after anti-TB chemotherapy without any neurological deficit or damage been observed.

## Conclusion

Tuberculous spondylitis is considered an extremely rare clinical entity known also as Pott’s disease. In the era of modern imaging modalities and effective anti-TB medication, TB appears to be a disease too frequently forgotten by clinicians and underemphasized by academic scholars. Misdiagnosis and delay in treatment are common events. A high degree of suspicion is necessary in order to avoid delays that may result in irreversible damage and high mortality rate. In patients presenting with chronic symptomatology of the lungs and suspicious radiological presentation, the clinicians should proceed to further clinical and laboratory tests, even if Mantoux test and sputum cultivation are negative. It is also suggested to administer treatment before definite diagnosis. Clinicians should consider Pott’s disease in the differential diagnosis of patients with back or shoulder pain and possible destructive vertebral lesions. Proper diagnosis and anti-TB treatment with or without surgery will result in cure.

## Abbreviations

ESR: erythrocyte sedimentation rate
LDH: lactate dehydrogenase
PCR: polymerase chain reaction
PLT: platelets
SGOT: serum glutamic oxaloacetic transaminase
SGPT: serum glutamic pyruvic transaminase
TB: tuberculosis
WBC: white blood cell
